# Awake Craniotomy in Africa: A Scoping Review of Literature and Proposed Solutions to Tackle Challenges

**DOI:** 10.1227/neu.0000000000002453

**Published:** 2023-03-24

**Authors:** Mohammad Mofatteh, Mohammad Sadegh Mashayekhi, Saman Arfaie, Amos Olufemi Adeleye, Edward Olaoluwa Jolayemi, Nathalie C. Ghomsi, Nathan A. Shlobin, Ahmed A. Morsy, Ignatius N. Esene, Tsegazeab Laeke, Ahmed K. Awad, Jason J. Labuschagne, Richard Ruan, Yared Nigusie Abebe, John Nute Jabang, Abiodun Idowu Okunlola, Umaru Barrie, Hervé Monka Lekuya, Ehanga Idi Marcel, Kantenga Dieu Merci Kabulo, Nourou Dine Adeniran Bankole, Idara J. Edem, Chibuikem A. Ikwuegbuenyi, Stephane Nguembu, Yvan Zolo, Mark Bernstein

**Affiliations:** * School of Medicine, Dentistry, and Biomedical Sciences, Queen's University Belfast, Belfast, UK;; ‡ Faculty of Medicine, University of British Columbia, Vancouver, British Columbia, Canada;; § Department of Neurology and Neurosurgery, McGill University, Montreal, Quebec, Canada;; ¶ Department of Surgery, College of Medicine, University of Ibadan, Ibadan, Nigeria;; # Neurosurgery Unit, Department of Surgery, Evercare Hospital Lagos, Nigeria;; ** Neurosurgery Department, Felix Houphouet Boigny Unversity Abidjan, Cote d’Ivoire;; †† Department of Neurological Surgery, Northwestern University Feinberg School of Medicine, Chicago, Illinois, USA;; ‡‡ Department of Neurosurgery, Zagazig University, Zagazig, Egypt;; §§ Neurosurgery Division, Faculty of Health Sciences, University of Bamenda, Bambili, Cameroon;; ǁǁ Neurosurgery Division, Department of Surgery, College of Health Sciences, Addis Ababa University, Addis Ababa, Ethiopia;; ¶¶ Faculty of Medicine, Ain-shams University, Cairo, Egypt;; ## Department of Neurosurgery, University of the Witwatersrand, Johannesburg, South Africa;; *** Division of Infectious Diseases and Vaccinology, School of Public Health, University of California, Berkeley, Berkeley, California, USA;; ††† Department of Neurosurgery, Haramaya University Hiwot Fana Comprehensive Specialized Hospital, Harar, Ethiopia;; ‡‡‡ Edward Francis Small Teaching Hospital, Banjul, Gambia;; §§§ Department of Surgery, Federal Teaching Hospital Ido Ekiti and Afe Babalola University, Ado Ekiti, Nigeria;; ǁǁǁ Department of Neurological Surgery, University of Texas Southwestern Medical Center, Dallas, Texas, USA;; ¶¶¶ Department of Neurosurgery, Makerere University/Mulago Hospital, Kampala, Uganda;; ### Department of Neurosurgery, College of Surgeons of East, Central and Southern Africa/Mulago Hospital, Kampala, Uganda;; **** Department of Neurosurgery, Jason Sendwe General Provincial Hospital, Lubumbashi, Democratic Republic of the Congo;; †††† Department of Neurosurgery, Hôpital Des Spécialités, WFNS Rabat Training Center For Young, African Neurosurgeons, Faculty of Medicine, Mohammed V University, Rabat, Morocco;; ‡‡‡‡ Department of Surgery, College of Human Medicine, Michigan State University, East Lansing, Michigan, USA;; §§§§ Research Department, Association of Future African Neurosurgeons, Yaounde, Cameroon;; ǁǁǁǁ Department of Neurosurgery, Higher Institute of Health Sciences, Université des Montagnes, Bangangté, Cameroon;; ¶¶¶¶ Global Surgery Division, University of Cape Town, Cape Town, South Africa;; #### Division of Neurosurgery, Department of Surgery, University of Toronto, University Health Network, Toronto, Ontario, Canada;; ***** Temmy Latner Center for Palliative Care, Mount Sinai Hospital, University of Toronto, Toronto, Ontario, Canada

**Keywords:** Africa, Anesthesia, Awake craniotomy, Brain mapping, Glioma, Global neurosurgery, Low- and middle-income countries, Tumors

## Abstract

Awake craniotomy (AC) is a common neurosurgical procedure for the resection of lesions in eloquent brain areas, which has the advantage of avoiding general anesthesia to reduce associated complications and costs. A significant resource limitation in low- and middle-income countries constrains the usage of AC.

**OBJECTIVE:**

To review the published literature on AC in African countries, identify challenges, and propose pragmatic solutions by practicing neurosurgeons in Africa.

**METHODS:**

We conducted a scoping review under Preferred Reporting Items for Systematic Reviews and Meta-Analysis-Scoping Review guidelines across 3 databases (PubMed, Scopus, and Web of Science). English articles investigating AC in Africa were included.

**RESULTS:**

Nineteen studies consisting of 396 patients were included. Egypt was the most represented country with 8 studies (42.1%), followed by Nigeria with 6 records (31.6%). Glioma was the most common lesion type, corresponding to 120 of 396 patients (30.3%), followed by epilepsy in 71 patients (17.9%). Awake-awake-awake was the most common protocol used in 7 studies (36.8%). Sixteen studies (84.2%) contained adult patients. The youngest reported AC patient was 11 years old, whereas the oldest one was 92. Nine studies (47.4%) reported infrastructure limitations for performing AC, including the lack of funding, intraoperative monitoring equipment, imaging, medications, and limited human resources.

**CONCLUSION:**

Despite many constraints, AC is being safely performed in low-resource settings. International collaborations among centers are a move forward, but adequate resources and management are essential to make AC an accessible procedure in many more African neurosurgical centers.

**ABBREVIATIONS:**
AC: awake craniotomy
AED: antiepileptic drug
DES: direct electrical stimulation
EEG: electroencephalogram
GA: general anesthesia
HGG: high-grade glioma
LGG: low-grade glioma
LMIC: low- and middle-income countries
MDT: multidisciplinary team
NA: not applicable
NS: not specified
PONV: postoperative nausea and vomiting
PPE: personal protective equipment
PRISMA-ScR: Preferred Reporting Items for Systematic Reviews and Meta-Analysis-Scoping Review.

Recent global estimates indicate that although more than 22.6 million people suffer from disorders and injuries requiring the expertise of a neurosurgeon yearly, about 5 million individuals are unable to undergo the required neurosurgical treatments because of multiple factors, including resource limitations.^1^ This is particularly the case in low- and middle-income countries (LMIC) in Africa, where neurosurgical procedures are mainly performed under general anesthesia (GA), a more resource-intensive undertaking usually requiring postoperative hospitalization and rehabilitation.^2^ It is an unfortunate global economic reality that worldwide disparities in neurosurgical care can and unfortunately do result in preventable disabilities and deaths in many LMIC,^1^ a fact further affirmed by the revelation that neurological diseases are the leading cause of disability-adjusted life-years and the second leading cause of global mortality.^3^

Awake craniotomy (AC) is becoming a common neurosurgical procedure used in many advanced neurosurgical units to aid real-time mapping of eloquent brain areas to maximize lesion resection while minimizing postoperative neurological complications, and its use is beneficial within LMIC.^4^ AC is used for various indications, including epilepsy, tumors, arteriovenous malformations, aneurysms, and deep brain stimulation.^5-7^ Patients are sedated while awake for parts or all of the surgery duration, thereby bypassing some demands and complications often associated with GA, which includes endotracheal intubation, and the need for ventilators, arterial lines, catheters, and postoperative intensive care,^8^ and yielding better patient outcomes when compared with GA.^9,10^ Furthermore, AC can lower the long-term costs associated with neurosurgical procedures by reducing the hospitalization time, decreasing the requirement for extensive monitoring devices, shortening the recovery time after discharge, reducing morbidity, and preserving patients' employment because of low postoperative neurological deficits.^11-14^ Intuitively, these benefits should merit AC's use as a feasible procedure to be more frequently deployed in the LMIC just as it is regularly performed in the developed countries.^15,16^ However, several factors have acted as barriers that make its use relatively uncommon in LMIC.^17-19^

One of the questions that must be addressed in the discourse surrounding AC concerns itself with the limited data published from LMIC, especially in the Eastern, Central, and most of Western Africa, in treating patients. In this scoping review, we explore the present state of AC in Africa and identify key barriers along the way. We then propose practical solutions to overcome some of the challenges by relying on the meticulous analysis of some of the continent's practicing neurosurgeons. To our knowledge, this is the first review that accomplishes a synthesis of the published peer-reviewed literature on AC in Africa with the aforementioned goals in mind.

## METHODS

### Search Strategy

This scoping review was conducted based on the Preferred Reporting Items for Systematic Reviews and Meta-Analysis-Scoping Review (PRISMA-ScR).^20^ A population (African patients), concept (AC), and context (limited-resource setting) were established. PubMed, Scopus, and Web of Science databases were searched from inception to June 20th 2022 for relevant articles. Details of the search terms for each database are available in **Supplementary Table 1,** http://links.lww.com/NEU/D710. We did not use Boolean terms for Africa or individual countries to avoid the possibility of excluding relevant articles. Instead, we screened the affiliation of the listed authors for a more comprehensive search strategy. Articles were selected initially if at least 1 author was from Africa. Full-text articles were then read to establish whether the investigation was conducted in Africa.

### Inclusion and Exclusion Criteria

Articles were included if they met the following criteria: (1) original peer-reviewed articles with a digital objective identifier, (2) English only, (3) AC used as a neurosurgical procedure in Africa, (4) provided sufficient quantitative data and were accessible on databases used, and (5) articles involving human subjects only. The exclusion criteria were (1) articles that aggregated AC data on African and non-African countries without differentiating the Africa sources, (2) sufficient extractable data were not provided, and (3) studies that investigated neurosurgical interventions other than AC (eg, minicraniotomy^21^). Case-reports and letters to editors were excluded unless sufficient data were provided. Data were extracted and all calculations were done on Microsoft Excel (version 2016; Microsoft).

## RESULTS

### Overview of Literature Search Results

**FIGURE 1.**
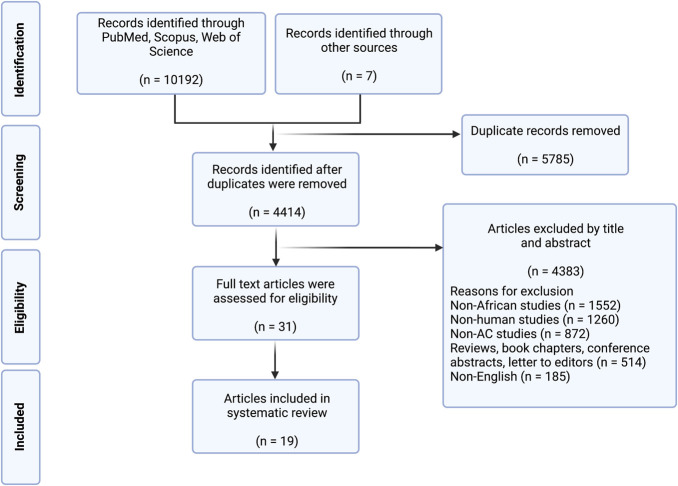
Preferred Reporting Items for Systematic Reviews and Meta-Analysis-Scoping Review flowchart demonstrating the search, screen, inclusion, and exclusion processes for this study.

**FIGURE 2.**
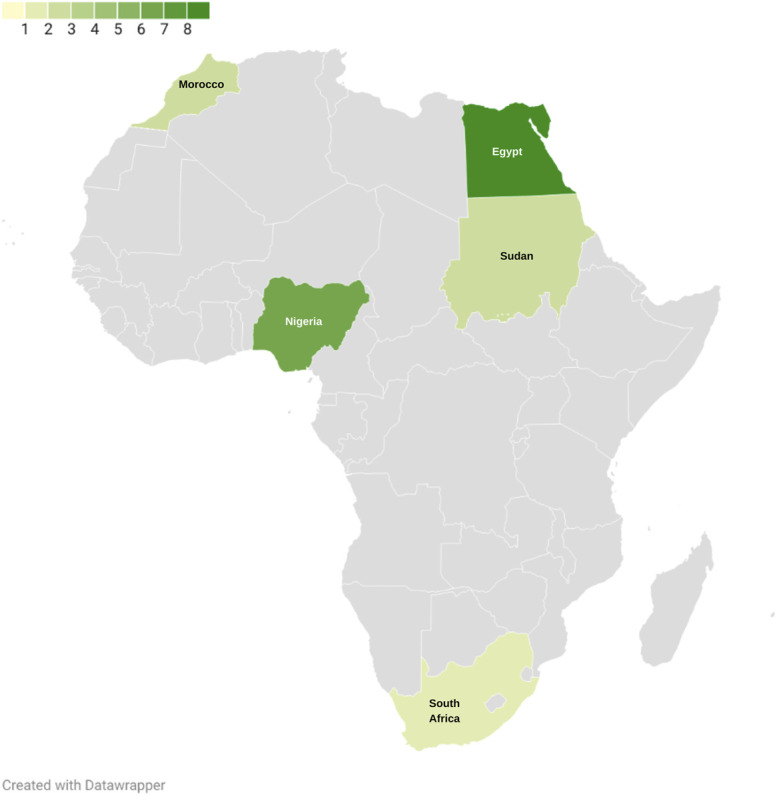
The continental distribution of included studies. Egypt, Nigeria, Sudan, Morocco, and South Africa are colored, and the heatmap shows publication number per country.

Details of our search results are shown in Figure 1. Nineteen studies were included: 8 (42.1%) were from Egypt,^22-29^ 6 (31.6%) from Nigeria,^30-35^ 2 each (10.5%) from Sudan^36,37^ and Morocco,^38,39^ and 1 study (5.26%) was from South Africa^40^ (**Supplementary Table 2,** http://links.lww.com/NEU/D711). The intracontinental distribution of these studies is also schematically represented (Figure 2).

AC was used for various surgical indications, including tumor, epilepsy, and arteriovenous malformation.^41-47^ All studies, except 4, reported AC on tumors only (78.9%).^22,23,26,29,31,32,34-40^ 1 study (5.3%) reported AC in epilepsy,^25^ whereas 3 studies (15.8%) reported other surgical indications in addition to tumor^24,30,33^ (**Supplementary Table 3,** http://links.lww.com/NEU/D712).

Although AC is an established technique, considerable differences exist between surgical approaches, regarding the anesthesia protocol and medication usage for sedation and analgesia.^4,8,11^ Awake-awake-awake was the most common AC protocol used in 7 studies (36.8%),^24,26,28,29,32,36,37^ followed by asleep-awake-asleep in 3 studies (15.8%)^31,39,40^ (**Supplementary Table 3,** http://links.lww.com/NEU/D712).

With some variations in different centers, propofol, remifentanil, and dexmedetomidine are frequently used medications in AC^48-50^; however, medication use varies between different centers. Eighteen studies (94.7%) reported the primary analgesic and sedation used in AC.^22-34,36-40^ Propofol/fentanyl combination was used for sedation/analgesia in 10 studies (52.6%)^22,24,26-31,36,37^ (**Supplementary Table 3,** http://links.lww.com/NEU/D712).

Sixteen studies (84.2%) provided information on operation time.^22-28,30-32,34-37,39,40^ The longest mean operation time was more than 237 minutes,^24^ whereas the shortest operation time was 1 hour^33^ (**Supplementary Table 3,** http://links.lww.com/NEU/D712).

### Awake Craniotomy in Adults and Pediatrics

In total, 396 patients were included in this review. Elbakry et al^25^ had the largest sample size, with 65 patients (16.4%), whereas the smallest sample size was in case-reports with 1 patient (0.25%) each.^34-36,38,40^ Apart from 2 studies (10.5%) that had a mixture of adults and pediatric patients,^28,30^ and 1 study (5.26%) that was on a child,^40^ all other studies (84.2%) had adult patient populations.^22-27,29,31-39^ The youngest reported AC patient was 11 years old,^40^ with the oldest being 92 years old,^33^ demonstrating the accessibility of AC for a wide age range. Relative to adults undergoing operations, AC is less widely used in children, likely due to case complexity, a higher risk of postoperative neurocognitive deficits, and a lower frequency of lesions in eloquent areas^51,52^ (Table 1).

**TABLE 1.**
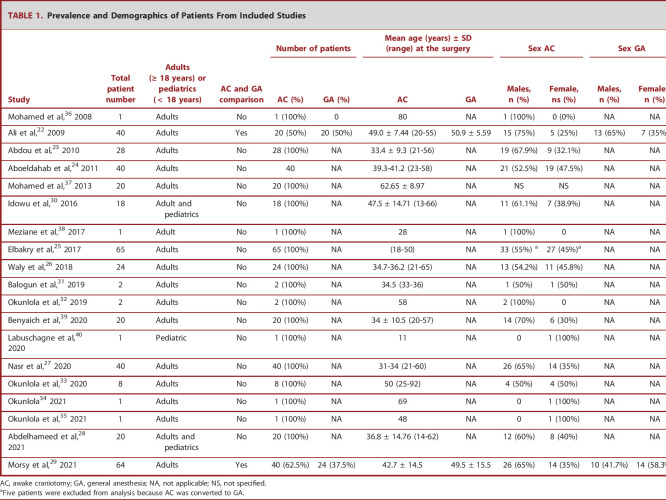
Prevalence and Demographics of Patients From Included Studies

				Number of patients		Mean age (years) ± SD (range) at the surgery		Sex AC		Sex GA	
Study	Total patient number	Adults (≥ 18 years) or pediatrics (< 18 years)	AC and GA comparison	AC (%)	GA (%)	AC	GA	Males, n (%)	Female, ns (%)	Males, n (%)	Females, n (%)
Mohamed et al,^36^ 2008	1	Adults	No	1 (100%)	0	80	NA	1 (100%)	0 (0%)	NA	NA
Ali et al,^22^ 2009	40	Adults	Yes	20 (50%)	20 (50%)	49.0 ± 7.44 (20-55)	50.9 ± 5.59	15 (75%)	5 (25%)	13 (65%)	7 (35%)
Abdou et al,^23^ 2010	28	Adults	No	28 (100%)	NA	33.4 ± 9.3 (21-56)	NA	19 (67.9%)	9 (32.1%)	NA	NA
Aboeldahab et al,^24^ 2011	40	Adults	No	40	NA	39.3-41.2 (23-58)	NA	21 (52.5%)	19 (47.5%)	NA	NA
Mohamed et al,^37^ 2013	20	Adults	No	20 (100%)	NA	62.65 ± 8.97	NA	NS	NS	NA	NA
Idowu et al,^30^ 2016	18	Adult and pediatrics	No	18 (100%)	NA	47.5 ± 14.71 (13-66)	NA	11 (61.1%)	7 (38.9%)	NA	NA
Meziane et al,^38^ 2017	1	Adult	No	1 (100%)	NA	28	NA	1 (100%)	0	NA	NA
Elbakry et al,^25^ 2017	65	Adults	No	65 (100%)	NA	(18-50)	NA	33 (55%) ^a^	27 (45%)^a^	NA	NA
Waly et al,^26^ 2018	24	Adults	No	24 (100%)	NA	34.7-36.2 (21-65)	NA	13 (54.2%)	11 (45.8%)	NA	NA
Balogun et al,^31^ 2019	2	Adults	No	2 (100%)	NA	34.5 (33-36)	NA	1 (50%)	1 (50%)	NA	NA
Okunlola et al,^32^ 2019	2	Adults	No	2 (100%)	NA	58	NA	2 (100%)	0	NA	NA
Benyaich et al,^39^ 2020	20	Adults	No	20 (100%)	NA	34 ± 10.5 (20-57)	NA	14 (70%)	6 (30%)	NA	NA
Labuschagne et al,^40^ 2020	1	Pediatric	No	1 (100%)	NA	11	NA	0	1 (100%)	NA	NA
Nasr et al,^27^ 2020	40	Adults	No	40 (100%)	NA	31-34 (21-60)	NA	26 (65%)	14 (35%)	NA	NA
Okunlola et al,^33^ 2020	8	Adults	No	8 (100%)	NA	50 (25-92)	NA	4 (50%)	4 (50%)	NA	NA
Okunlola^34^ 2021	1	Adults	No	1 (100%)	NA	69	NA	0	1 (100%)	NA	NA
Okunlola et al,^35^ 2021	1	Adults	No	1 (100%)	NA	48	NA	0	1 (100%)	NA	NA
Abdelhameed et al,^28^ 2021	20	Adults and pediatrics	No	20 (100%)	NA	36.8 ± 14.76 (14-62)	NA	12 (60%)	8 (40%)	NA	NA
Morsy et al,^29^ 2021	64	Adults	Yes	40 (62.5%)	24 (37.5%)	42.7 ± 14.5	49.5 ± 15.5	26 (65%)	14 (35%)	10 (41.7%)	14 (58.3%)

AC, awake craniotomy; GA, general anesthesia; NA, not applicable; NS, not specified.

a Five patients were excluded from analysis because AC was converted to GA.

Two studies (10.5%) compared AC with GA,^22,29^ and demonstrated better patient outcomes for AC patients compared with GA. For example, although 14 patients (70%) were admitted into intensive care unit (ICU) in the GA group (5 seizures, 4 brain edema, 3 delayed recoveries, and 2 agitations), only 2 AC patients (10%) had postoperative complications (1 seizure and 1 brain edema) requiring ICU care (*P* = .0004).^22^ The same group showed the hospitalization length was reduced from 8.15 ± 6.5 days in the GA cohort to 3.8 ± 4.15 days in the AC group (*P* = .05).^22^ Furthermore, another study illustrated the advantage of AC over GA by reporting less permanent postoperative neurological deficits in AC patients (5%) vs those undergoing GA (8.3%) (*P* = .59)^29^ (Table 1).

### Lesion Characteristics

Sixteen studies (84.2%) clarified lesion types.^22,24,25,28-40^ Glioma in 120 patients (30.3%) was the most common, followed by epilepsy in 71 (17.9%) and nonglioma tumor in 36 (9.10%) (Table 2).

**TABLE 2.**
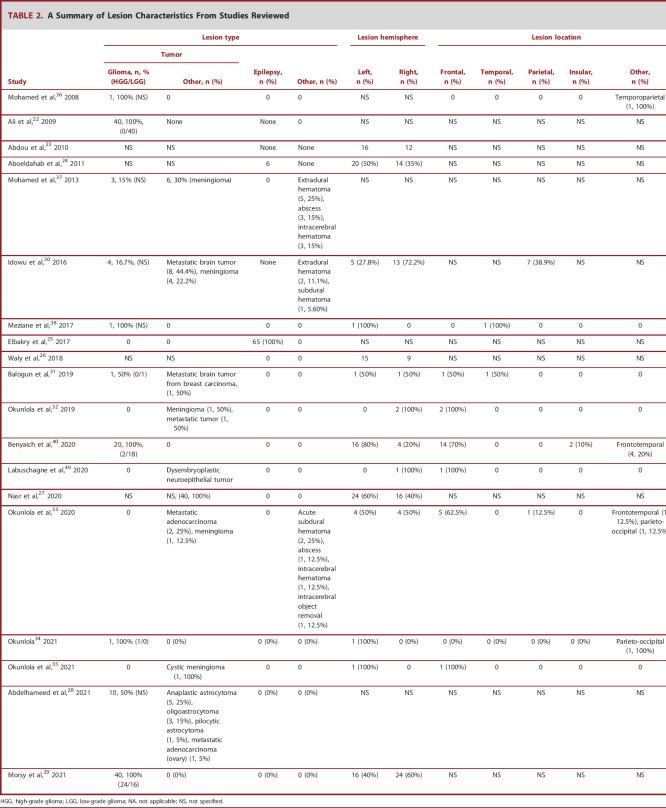
A Summary of Lesion Characteristics From Studies Reviewed

	Lesion type				Lesion hemisphere		Lesion location				
	Tumor		Epilepsy, n (%)	Other, n (%)	Left, n (%)	Right, n (%)	Frontal, n (%)	Temporal, n (%)	Parietal, n (%)	Insular, n (%)	Other, n (%)
Study	Glioma, n, % (HGG/LGG)	Other, n (%)									
Mohamed et al,^36^ 2008	1, 100% (NS)	0	0	0	NS	NS	0	0	0	0	Temporoparietal (1, 100%)
Ali et al,^22^ 2009	40, 100%, (0/40)	None	None	0	NS	NS	NS	NS	NS	NS	NS
Abdou et al,^23^ 2010	NS	NS	None	None	16	12	NS	NS	NS	NS	NS
Aboeldahab et al,^24^ 2011	NS	NS	6	None	20 (50%)	14 (35%)	NS	NS	NS	NS	NS
Mohamed et al,^37^ 2013	3, 15% (NS)	6, 30% (meningioma)	0	Extradural hematoma (5, 25%), abscess (3, 15%), intracerebral hematoma (3, 15%)	NS	NS	NS	NS	NS	NS	NS
Idowu et al,^30^ 2016	4, 16.7%, (NS)	Metastatic brain tumor (8, 44.4%), meningioma (4, 22.2%)	None	Extradural hematoma (2, 11.1%), subdural hematoma (1, 5.60%)	5 (27.8%)	13 (72.2%)	NS	NS	7 (38.9%)	NS	NS
Meziane et al,^38^ 2017	1, 100% (NS)	0	0	0	1 (100%)	0	0	1 (100%)	0	0	0
Elbakry et al,^25^ 2017	0	0	65 (100%)	0	NS	NS	NS	NS	NS	NS	NS
Waly et al,^26^ 2018	NS	NS	0	0	15	9	NS	NS	NS	NS	NS
Balogun et al,^31^ 2019	1, 50% (0/1)	Metastatic brain tumor from breast carcinoma, (1, 50%)	0	0	1 (50%)	1 (50%)	1 (50%)	1 (50%)	0	0	0
Okunlola et al,^32^ 2019	0	Meningioma (1, 50%), metastatic tumor (1, 50%)	0	0	0	2 (100%)	2 (100%)	0	0	0	0
Benyaich et al,^40^ 2020	20, 100%, (2/18)	0	0	0	16 (80%)	4 (20%)	14 (70%)	0	0	2 (10%)	Frontotemporal (4, 20%)
Labuschagne et al,^40^ 2020	0	Dysembryoplastic neuroepithelial tumor	0	0	0	1 (100%)	1 (100%)	0	0	0	0
Nasr et al,^27^ 2020	NS	NS, (40, 100%)	0	0	24 (60%)	16 (40%)	NS	NS	NS	NS	NS
Okunlola et al,^33^ 2020	0	Metastatic adenocarcinoma (2, 25%), meningioma (1, 12.5%)	0	Acute subdural hematoma (2, 25%), abscess (1, 12.5%), intracerebral hematoma (1, 12.5%), intracerebral object removal (1, 12.5%)	4 (50%)	4 (50%)	5 (62.5%)	0	1 (12.5%)	0	Frontotemporal (1, 12.5%), parieto-occipital (1, 12.5%)
Okunlola^34^ 2021	1, 100% (1/0)	0 (0%)	0 (0%)	0 (0%)	1 (100%)	0 (0%)	0 (0%)	0 (0%)	0 (0%)	0 (0%)	Parieto-occipital (1, 100%)
Okunlola et al,^35^ 2021	0	Cystic meningioma (1, 100%)	0	0	1 (100%)	0	1 (100%)	0	0	0	0
Abdelhameed et al,^28^ 2021	10, 50% (NS)	Anaplastic astrocytoma (5, 25%), oligoastrocytoma (3, 15%), pilocytic astrocytoma (1, 5%), metastatic adenocarcinoma (ovary) (1, 5%)	0 (0%)	0 (0%)	NS	NS	NS	NS	NS	NS	NS
Morsy et al,^29^ 2021	40, 100% (24/16)	0 (0%)	0 (0%)	0 (0%)	16 (40%)	24 (60%)	NS	NS	NS	NS	NS

HGG, high-grade glioma; LGG, low-grade glioma; NA, not applicable; NS, not specified.

Thirteen studies (68.4%) specified the preoperative diagnostic imaging methods for localizing the lesions.^22,26,28-31,33-37,39,40^ MRI was the most common method used in 8 studies (42.1%) for preoperative localization,^26,31,34-37,39,40^ whereas computerized tomography (CT) and MRI were used in 4 studies (21.1%).^22,28,30,33^ (**Supplementary Table 4,** http://links.lww.com/NEU/D713).

Nine studies (47.4%) reported the duration of hospitalization,^22,23,28,30-33,39^ with the longest being 5 to 11 days.^33^ Notably, same-day discharge was reported for 2 patients^31^ (**Supplementary Table 4,** http://links.lww.com/NEU/D713).

### Intraoperative Description of AC

The primary purpose of AC is to resect lesions in visual, language, sensory, motor, and other eloquent areas.^4^ Nine studies (47.4%) specified their lesion area.^22,26,28,29,32,34,38-40^ Four studies (21.1%) had motor area lesions,^29,32,34,40^ whereas motor and language area lesions were reported in 4 other studies (21.1%)^22,28,38,39^ (**Supplementary Table 5,** http://links.lww.com/NEU/D714).

Eloquent area mapping is an integral part of the AC program, influencing the extent of resection and, thereby, prognosis.^53,54^ Direct electrical stimulation (DES) is the gold standard for identifying eloquent areas to maximize lesion resection and reduce new neurological deficits.^55-57^ Relying solely on preoperative clinical information to determine the eloquent areas is associated with significant morbidity.^58^ Only 6 studies (31.6%) used DES.^26-29,39,40^ Others relied on continuous monitoring of patients' speech and motor functions,^22,36,37^ because of a lack of stimulator devices, which is a common problem in LMIC^13^ (**Supplementary Table 5,** http://links.lww.com/NEU/D714).

Conversion to GA can complicate AC, rendering the procedure a failure, for many reasons, including uncontrolled intraoperative seizure and lack of patient tolerance.^59,60^ Seven AC patients (1.77%) from 3 studies (15.8%) had conversion to GA^25,26,28^ (**Supplementary Table 5,** http://links.lww.com/NEU/D714).

### Constraints to AC in Low-Resource Centers

Lack of appropriate infrastructure, shortage of surgical, nursing, and anesthesia staff, prolonged waiting time, and the quality of training have been considered some of the constraints of neurosurgical care.^61^ The safety and feasibility of AC in low-resource settings where modern and expensive technologies, such as functional and intraoperative MRI, intraoperative cortical mapping, and electrophysiology, are not available can add significant challenges.^22^ Nine studies (47.4%) reported infrastructure limitations as an obstacle to performing AC operations,^22,26,28,30-34,39^ with 1 study noting that the lack of head pins at their hospital hindered AC procedures^30^ (Table 3).

**TABLE 3.**
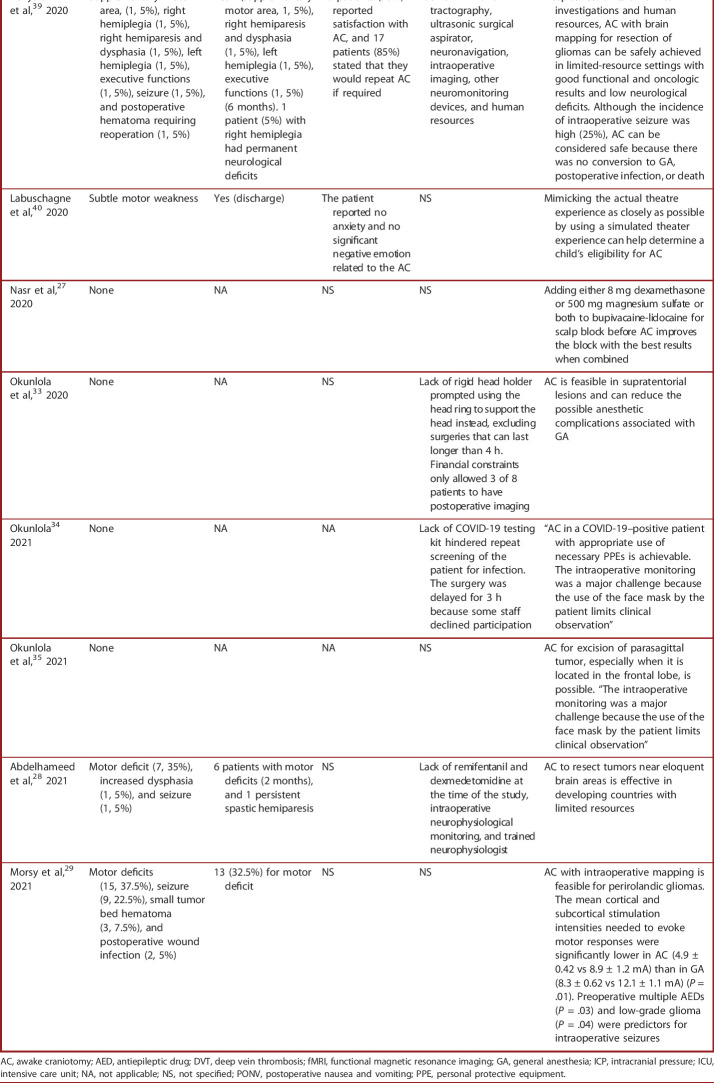
A Summary of Patient Outcomes and Limitations

Study	Postoperative complications (n, %)	Postoperative complications resolve (duration)	Patient satisfaction	Infrastructure limitations stated	Main outcomes reported
Mohamed et al,^36^ 2008	None	NA	NS	NS	AC is feasible in low-resource settings to provide good prognosis and reduce costs without compromising patients' safety
Ali et al,^22^ 2009	Aphasia (1, 5%), hemiparesis (1, 5%), and ICU admission (2)	Yes (4 wk to 6 mo)	NS	Lack of fMRI, intraoperative mapping, ICP monitoring, and neurophysiologist	90% of AC compared with 40% of GA patients had improved neurological deficits (*P* = 0). However, this difference was not statistically significant at 6 months of follow-up. Mean hospitalization was reduced significantly in the AC group (*P* = .05)
Abdou et al,^23^ 2010	Neurological deficits (4, 14.3%), seizure (3, 10.7%), and nausea and vomiting (2, 7.14%)	Yes (NS)	78.6% of patients were satisfied	NS	Conscious sedation during AC using ketofol infusion mixture in 1:1 ratio is safe and efficient with minor respiratory and hemodynamic events and rapid smooth recovery
Aboeldahab et al,^24^ 2011	PONV, seizure, and electrolyte disturbances (NS)	NS	NS	NS	Appropriate selection of patients, proper positioning, and adoption of the conscious sedation technique with voluntary hyperventilation makes mannitol usage unnecessary during AC
Mohamed et al,^37^ 2013	Hemiparesis (2, 10%)	Yes (3 wk)	NS	NS	Maximal tumor resection with minimal impairment of neurological functions can be achieved using AC in low-resource settings
Idowu et al,^30^ 2016	Aphasia (1, 5.5%), hypotension (1, 5.5%), DVT (1, 5.5%), and pulmonary edema (1, 5.5%)	NS	NS	Lack of head pins for patient positioning	AC is a well-tolerated procedure with a low rate of complications in low-resource settings
Meziane et al,^38^ 2017	None	NA	NS	NS	Psychological preparation, a reasonable choice of anesthetic techniques and agents, and continuous team communication are key reasons for successful outcomes in AC patients
Elbakry et al,^25^ 2017	NS	NS	Patients' satisfaction was high (6.33-6.45 of 10, with 10 being the most satisfied)	NS	Although the propofol-dexmedetomidine combination is as effective as the propofol-remifentanil combination, it has fewer side effects during AC for epilepsy
Waly et al,^26^ 2018	None	NA	19 patients (79.16%) were satisfied	Medications such as dexmedetomidine were not available	“Both dexmedetomidine and midazolam were safe and efficient during AC. Midazolam had a higher success rate, lower incidence of intraoperative seizures, and higher incidence of amnesia. Dexmedetomidine had more rapid recovery”
Balogun et al,^31^ 2019	None	NA	100% satisfaction with AC and same-day discharge	Lack of intraoperative brain mapping, histology facility, day-care admission unit, and ICU. Financial constraints for postoperative MRI delayed radiation therapy	AC is safe and feasible in a low-resource setting and resulted in same-day discharge to reduce brain surgery costs without causing any new postoperative neurological deficits
Okunlola et al,^32^ 2019	None	NA	NS	Lack of rigid head holder, neurophysiological monitor, and neuroimaging facilities. Limited mechanical ventilator. No access to neuroanesthetist with experience in AC	AC should be considered as an option in resource-poor settings in selected patients to reduce operative morbidity and pressure on the limited human and material resources
Benyaich et al,^39^ 2020	Supplementary motor area, (1, 5%), right hemiplegia (1, 5%), right hemiparesis and dysphasia (1, 5%), left hemiplegia (1, 5%), executive functions (1, 5%), seizure (1, 5%), and postoperative hematoma requiring reoperation (1, 5%)	Yes - (supplementary motor area, 1, 5%), right hemiparesis and dysphasia (1, 5%), left hemiplegia (1, 5%), executive functions (1, 5%) (6 months). 1 patient (5%) with right hemiplegia had permanent neurological deficits	18 patients (90%) reported satisfaction with AC, and 17 patients (85%) stated that they would repeat AC if required	Lack of fMRI and tractography, ultrasonic surgical aspirator, neuronavigation, intraoperative imaging, other neuromonitoring devices, and human resources	Despite restraints on clinical investigations and human resources, AC with brain mapping for resection of gliomas can be safely achieved in limited-resource settings with good functional and oncologic results and low neurological deficits. Although the incidence of intraoperative seizure was high (25%), AC can be considered safe because there was no conversion to GA, postoperative infection, or death
Labuschagne et al,^40^ 2020	Subtle motor weakness	Yes (discharge)	The patient reported no anxiety and no significant negative emotion related to the AC	NS	Mimicking the actual theatre experience as closely as possible by using a simulated theater experience can help determine a child's eligibility for AC
Nasr et al,^27^ 2020	None	NA	NS	NS	Adding either 8 mg dexamethasone or 500 mg magnesium sulfate or both to bupivacaine-lidocaine for scalp block before AC improves the block with the best results when combined
Okunlola et al,^33^ 2020	None	NA	NS	Lack of rigid head holder prompted using the head ring to support the head instead, excluding surgeries that can last longer than 4 h. Financial constraints only allowed 3 of 8 patients to have postoperative imaging	AC is feasible in supratentorial lesions and can reduce the possible anesthetic complications associated with GA
Okunlola^34^ 2021	None	NA	NA	Lack of COVID-19 testing kit hindered repeat screening of the patient for infection. The surgery was delayed for 3 h because some staff declined participation	“AC in a COVID-19–positive patient with appropriate use of necessary PPEs is achievable. The intraoperative monitoring was a major challenge because the use of the face mask by the patient limits clinical observation”
Okunlola et al,^35^ 2021	None	NA	NA	NS	AC for excision of parasagittal tumor, especially when it is located in the frontal lobe, is possible. “The intraoperative monitoring was a major challenge because the use of the face mask by the patient limits clinical observation”
Abdelhameed et al,^28^ 2021	Motor deficit (7, 35%), increased dysphasia (1, 5%), and seizure (1, 5%)	6 patients with motor deficits (2 months), and 1 persistent spastic hemiparesis	NS	Lack of remifentanil and dexmedetomidine at the time of the study, intraoperative neurophysiological monitoring, and trained neurophysiologist	AC to resect tumors near eloquent brain areas is effective in developing countries with limited resources
Morsy et al,^29^ 2021	Motor deficits (15, 37.5%), seizure (9, 22.5%), small tumor bed hematoma (3, 7.5%), and postoperative wound infection (2, 5%)	13 (32.5%) for motor deficit	NS	NS	AC with intraoperative mapping is feasible for perirolandic gliomas. The mean cortical and subcortical stimulation intensities needed to evoke motor responses were significantly lower in AC (4.9 ± 0.42 vs 8.9 ± 1.2 mA) than in GA (8.3 ± 0.62 vs 12.1 ± 1.1 mA) (*P* = .01). Preoperative multiple AEDs (*P* = .03) and low-grade glioma (*P* = .04) were predictors for intraoperative seizures

AC, awake craniotomy; AED, antiepileptic drug; DVT, deep vein thrombosis; fMRI, functional magnetic resonance imaging; GA, general anesthesia; ICP, intracranial pressure; ICU, intensive care unit; NA, not applicable; NS, not specified; PONV, postoperative nausea and vomiting; PPE, personal protective equipment.

Optimal anesthesia techniques should achieve analgesia and sedation while preventing side effects such as nausea, vomiting, and seizures.^62^ Lack of access to special anesthetic medications, including remifentanil and dexmedetomidine, equipment required for neurophysiological monitoring and brain mapping, and the presence of an experienced team of neurosurgeons and anesthetists are some of the other limiting factors for carrying out AC in LMIC.^28^

Balogun et al^31^ in Nigeria did not have access to histology and molecular profiling facilities; therefore, they were unable to specify the tumor subtype with certainty. Furthermore, they reported financial constraints for postoperative MRIs delaying the radiation therapy initiation to the tumor bed, and whole-brain radiation therapy since health care in their setting is predominantly financed “out-of-pocket” by the patient.^31^ Benyaich et al,^39^ in their study conducted in Morocco, specified that lack of functional MRI and tractography, ultrasonic surgical aspirator, neuronavigation, intraoperative imaging, other neuromonitoring devices, and human resources as the main barriers at their center.

Safe AC requires the cooperation of the neurosurgical and anesthesia teams to maintain the patient awake during the resection, and monitor the patient's responses and development of new neurological deficits.^62^ Although other professionals may not be required at all times, Benyaich et al^39^ reported that neuropsychologists, speech therapists, and neurophysiologists were present for the first 2 procedures (10%) during the establishment of the programs in Morocco with the help of an experienced team from Europe. Thereafter, adaptations to the AC program were required to suit accessible resources, and only neurosurgeons and anesthesiologists with episodic participation of a neurologist with neuropsychological training were involved in the AC procedures.^39^

### Cultural and Religious Considerations

Local cultural values and expectations have been shown to influence patients' health-seeking behavior.^31,63^ For example, Benyaich et al^39^ reported a mean duration of 17 ± 4.9 months from the onset of symptoms to admission to the neurosurgical department, attributing such delays to cultural belief, such as the association of seizure—as it was the most common presenting symptom in 70% of their cases—with a social stigma of being possessed by demonical power. Furthermore, patients' preference to consult traditional healers without any medical training can also delay medical consultation.^64^ Even when patients seek medical consultation, lack of healthcare resources in many areas can delay treatment. For example, a lower ratio of MRI units per capita delays patient investigations.^65^ Such hindrances cause patients to present at advanced stages of the disease, therefore making their enrollment in the AC program challenging.^39^

## DISCUSSION

AC with brain mapping is a safe and cost-effective neurosurgical procedure enabling maximal lesion resection in eloquent brain areas while minimizing neurological complications, thereby providing an excellent alternative to craniotomy under GA.^55,66-68^

One of the advantages of AC over GA is the shorter hospitalization to save the hospital and human resources, particularly in low-resource settings, reduce postoperative complications, such as infection, and result in increased patient satisfaction.^8,66,69-72^ Although admission to ICU after brain surgery is a routine procedure in developed countries, which enables close monitoring and early detection of complications, establishing and maintaining neuro-ICU can be resource-intensive and costly in low-resource settings.^31,73-77^ If stringent patient inclusion criteria, including preoperative functional status of the patient, tumor location, brain edema, social support at home, and accessibility to hospital for readmission, are considered, AC patients can be discharged on the same or next day.^69,78,79^ Early discharge can be more suitable for both patients and the healthcare system.^78,80-83^ AC can also accelerate return to work to enable patients to continue with their employment,^84^ given that social support for disabled patients is scarce in Africa.^39^

It should be noted that postsurgical care at home and in the community requires reliable infrastructure and appropriate living conditions, which are not consistently available in Africa.^66^ For example, ambulances are largely absent in low-resource settings, and patients rely primarily on family and caregivers for transportation to and from the hospital, narrowing the spectrum of patients who can be offered outpatient surgical procedures.^31^ Therefore, we emphasize that biopsychosocial conditions should be considered when selecting patients for AC.

AC is a complex procedure relying on intraoperative brain mapping, imaging and neuromonitoring, specific anesthetic medications, and trained teams, which are not readily available in LMIC.^85-88^ Intraoperative functional mapping during AC can reduce the incidence of postoperative deficits from 13%- to 27.5% to less than 2%.^89^ Nevertheless, DES of cortical and subcortical areas to identify eloquent areas is not feasible in all centers, especially those in Africa, because of a lack of infrastructure and financial constraints. Recent studies also suggest the assessment of intraoperative neurophysiological parameters such as electrocorticography and electromyography, as well as using artificial intelligence, augmented and virtual reality in AC.^90-93^ The utilization of such equipment and technologies increases the cost of the procedure and is not readily available in many neurosurgery departments in Africa.^39,94,95^ However, such technological resources are not absolutely necessary.^18^

Best patient outcomes are achieved in neurosurgical centers with a multidisciplinary team with extensive experience in AC.^4,15,53,96^ Successful AC programs require a team of neurosurgeons, anesthesiologists, neurologists, neurophysiologists, neuropsychiatrists, specialized nurses, and speech therapists during preoperative, intraoperative, and postoperative phases of the procedure.^4^ Constraints in human resources have prompted neurosurgical colleagues from Africa to adapt AC programs with minimum staff where the intraoperative patient evaluation is safely conducted by neurosurgeons and anesthesiologists alone, with the occasional participation of neurologists who have neuropsychological training.^95^

Knowledge transfer can be used to initiate AC practice with minimum requirements. Enhancing global collaboration between centers with experience in performing AC in high-income countries is required to train competent staff and transfer knowledge for the sustainable establishment of AC centers, followed by supporting equipment required for safely performing operations. An example of the global effort to train teams needed to initiate AC in LMIC was reported in 6 neurosurgical centers in Nigeria, Ghana, Indonesia, and China.^13^ In-person visits and establishing partnerships can be supported longitudinally by internet-based content between neurosurgical centers experienced in AC and those in Africa.^18,97-99^ This can be sustained by knowledge transfer and collaboration within the continent between centers more experienced in AC with those that plan to initiate such programs.

Africa is a vast continent comprising 54 countries; however, AC studies included in this review were from 5 countries (9.26%) only. Although such findings can highlight the disparity that exists within the continent, an alternative explanation can be the lack of peer-reviewed publications on AC from African centers that were not represented.

**FIGURE 3.**
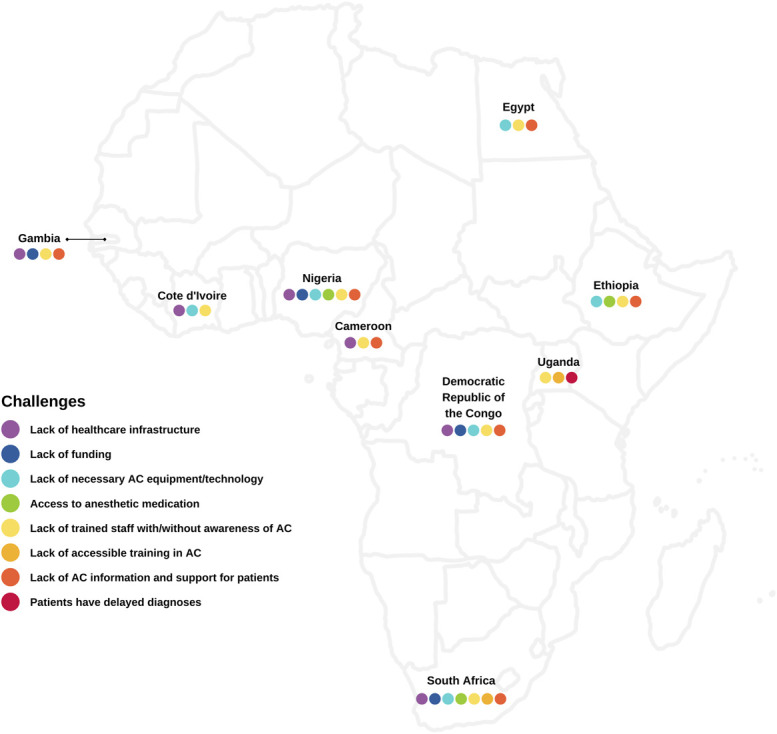
A summary of challenges identified in representative African countries to perform safe AC. AC, awake craniotomy.

**FIGURE 4.**
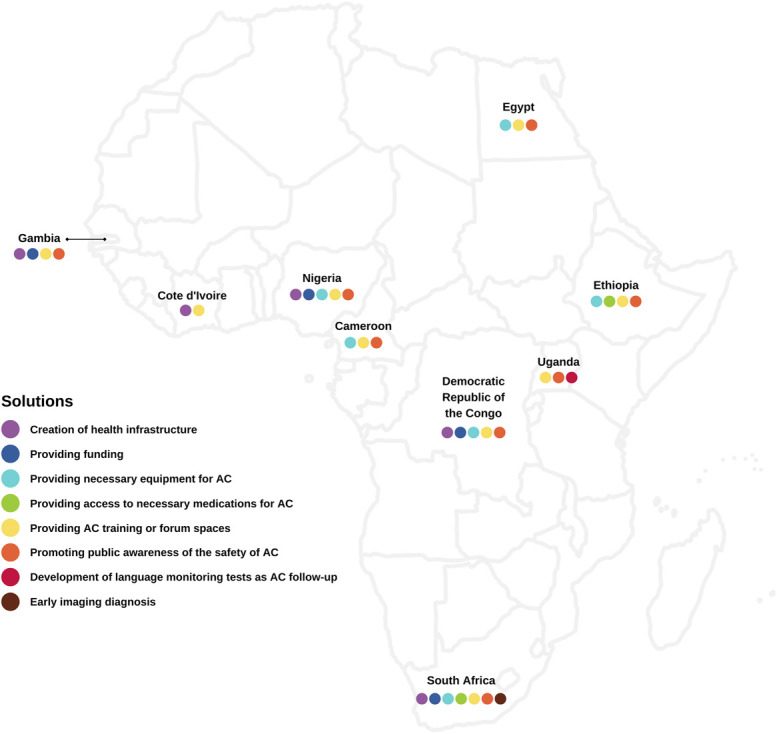
An overview of solutions suggested to tackle challenges associated with performing safe AC in different African countries. AC, awake craniotomy.

Based on the current findings as well as our experience of performing AC in LMIC, we highlight challenges associated with AC in Africa and propose pragmatic solutions to overcome them. We believe implementing these solutions can facilitate the usage of AC in different hospitals across Africa (Table 4). Furthermore, the challenges and magnitude of work that needs to be done by each Ministry of Health and neurosurgeons in representative African countries to initiate and sustain AC as a safe undertaking are illustrated in Figures 3 and 4.

**TABLE 4.**
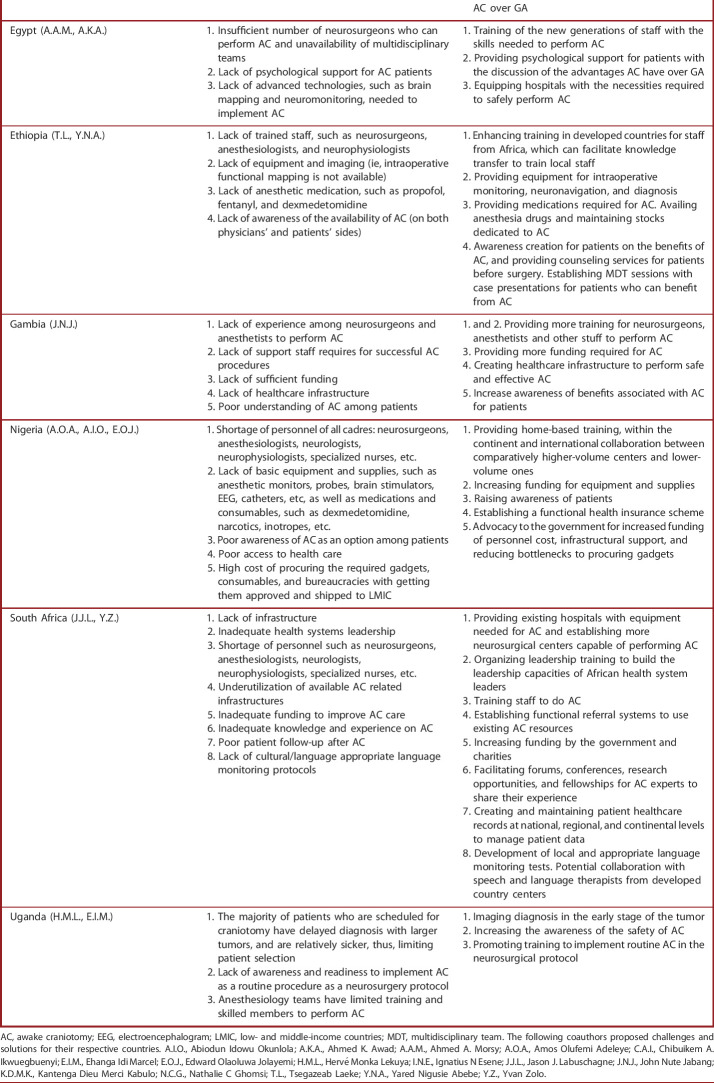
Challenges Identified and Proposed Solutions to Overcome Challenges of Practicing AC in Africa

Country (Initials of Neurosurgeons and Physicians Who Suggested Challenges and Solutions)	Challenges	Proposed solutions
Cameroon (I.N.E., C.A.I., N.C.G.)	1. Lack of access to intraoperative neuromonitoring and other infrastructure 2. Lack of access to specialized skills for using neuromonitoring 3. Inappropriate health-seeking behavior, especially regarding neurosurgical care	1. Investing in modern instruments 2. Providing subspecialized training in AC for young neurosurgeons 3. Promoting public awareness of neurosurgical care
Cote d’Ivoire (N.C.G.)	1. Inadequate healthcare infrastructure 2. Inadequate technology platform (ie, no functional MRI is present in the country) 3. Lack of trained neurosurgeons, anesthesiologists, neuropsychologists, and psychiatrists	1. Investing in modern infrastructures 2. Provide equipment required for specialized procedures 3. Increasing the workforce by expanding training
Democratic Republic of the Congo (K.D.M.K.)	1. Lack of equipment such as intraoperative MRI and electroneurophysiology 2. Inadequate number of neurosurgeons (ie, 16 neurosurgeons are serving a population of 9.5 million people) 3. Lack of sufficient funding 4. Lack of healthcare infrastructure 5. Misconception of the technique by patients and poor acceptance of neurosurgical procedures by Congolese population	1. Investing in acquiring specific instruments and devices by the government to help neurosurgeons perform AC in university teaching hospitals 2. Encouraging young neurosurgeons to pursue AC subspecialties 3. Providing more funding required to initiate and maintain successful AC program 4. Creating healthcare infrastructure 5. Providing psychological support for patients and organizing sessions to inform patient about the advantages of neurosurgical procedures such as AC over GA
Egypt (A.A.M., A.K.A.)	1. Insufficient number of neurosurgeons who can perform AC and unavailability of multidisciplinary teams 2. Lack of psychological support for AC patients 3. Lack of advanced technologies, such as brain mapping and neuromonitoring, needed to implement AC	1. Training of the new generations of staff with the skills needed to perform AC 2. Providing psychological support for patients with the discussion of the advantages AC have over GA 3. Equipping hospitals with the necessities required to safely perform AC
Ethiopia (T.L., Y.N.A.)	1. Lack of trained staff, such as neurosurgeons, anesthesiologists, and neurophysiologists 2. Lack of equipment and imaging (ie, intraoperative functional mapping is not available) 3. Lack of anesthetic medication, such as propofol, fentanyl, and dexmedetomidine 4. Lack of awareness of the availability of AC (on both physicians' and patients' sides)	1. Enhancing training in developed countries for staff from Africa, which can facilitate knowledge transfer to train local staff 2. Providing equipment for intraoperative monitoring, neuronavigation, and diagnosis 3. Providing medications required for AC. Availing anesthesia drugs and maintaining stocks dedicated to AC 4. Awareness creation for patients on the benefits of AC, and providing counseling services for patients before surgery. Establishing MDT sessions with case presentations for patients who can benefit from AC
Gambia (J.N.J.)	1. Lack of experience among neurosurgeons and anesthetists to perform AC 2. Lack of support staff requires for successful AC procedures 3. Lack of sufficient funding 4. Lack of healthcare infrastructure 5. Poor understanding of AC among patients	1. and 2. Providing more training for neurosurgeons, anesthetists and other stuff to perform AC 3. Providing more funding required for AC 4. Creating healthcare infrastructure to perform safe and effective AC 5. Increase awareness of benefits associated with AC for patients
Nigeria (A.O.A., A.I.O., E.O.J.)	1. Shortage of personnel of all cadres: neurosurgeons, anesthesiologists, neurologists, neurophysiologists, specialized nurses, etc. 2. Lack of basic equipment and supplies, such as anesthetic monitors, probes, brain stimulators, EEG, catheters, etc, as well as medications and consumables, such as dexmedetomidine, narcotics, inotropes, etc. 3. Poor awareness of AC as an option among patients 4. Poor access to health care 5. High cost of procuring the required gadgets, consumables, and bureaucracies with getting them approved and shipped to LMIC	1. Providing home-based training, within the continent and international collaboration between comparatively higher-volume centers and lower-volume ones 2. Increasing funding for equipment and supplies 3. Raising awareness of patients 4. Establishing a functional health insurance scheme 5. Advocacy to the government for increased funding of personnel cost, infrastructural support, and reducing bottlenecks to procuring gadgets
South Africa (J.J.L., Y.Z.)	1. Lack of infrastructure 2. Inadequate health systems leadership 3. Shortage of personnel such as neurosurgeons, anesthesiologists, neurologists, neurophysiologists, specialized nurses, etc. 4. Underutilization of available AC related infrastructures 5. Inadequate funding to improve AC care 6. Inadequate knowledge and experience on AC 7. Poor patient follow-up after AC 8. Lack of cultural/language appropriate language monitoring protocols	1. Providing existing hospitals with equipment needed for AC and establishing more neurosurgical centers capable of performing AC 2. Organizing leadership training to build the leadership capacities of African health system leaders 3. Training staff to do AC 4. Establishing functional referral systems to use existing AC resources 5. Increasing funding by the government and charities 6. Facilitating forums, conferences, research opportunities, and fellowships for AC experts to share their experience 7. Creating and maintaining patient healthcare records at national, regional, and continental levels to manage patient data 8. Development of local and appropriate language monitoring tests. Potential collaboration with speech and language therapists from developed country centers
Uganda (H.M.L., E.I.M.)	1. The majority of patients who are scheduled for craniotomy have delayed diagnosis with larger tumors, and are relatively sicker, thus, limiting patient selection 2. Lack of awareness and readiness to implement AC as a routine procedure as a neurosurgery protocol 3. Anesthesiology teams have limited training and skilled members to perform AC	1. Imaging diagnosis in the early stage of the tumor 2. Increasing the awareness of the safety of AC 3. Promoting training to implement routine AC in the neurosurgical protocol

AC, awake craniotomy; EEG, electroencephalogram; LMIC, low- and middle-income countries; MDT, multidisciplinary team. The following coauthors proposed challenges and solutions for their respective countries. A.I.O., Abiodun Idowu Okunlola; A.K.A., Ahmed K. Awad; A.A.M., Ahmed A. Morsy; A.O.A., Amos Olufemi Adeleye; C.A.I., Chibuikem A. Ikwuegbuenyi; E.I.M., Ehanga Idi Marcel; E.O.J., Edward Olaoluwa Jolayemi; H.M.L., Hervé Monka Lekuya; I.N.E., Ignatius N Esene; J.J.L., Jason J. Labuschagne; J.N.J., John Nute Jabang; K.D.M.K., Kantenga Dieu Merci Kabulo; N.C.G., Nathalie C Ghomsi; T.L., Tsegazeab Laeke; Y.N.A., Yared Nigusie Abebe; Y.Z., Yvan Zolo.

### Limitations

Our scoping review is subject to some limitations. Reviewed papers were published in English, and there was heterogeneity in the articles reviewed. Ten studies (52.6%) included in this review, with a total of 113 patients (28.5%), were retrospective, which can affect the level of the evidence available and the strength of analyses. In addition, the number of patients in each study and the data collection period varied widely. Furthermore, studies included were from 5 African countries, representing only 9.26% of African countries. A multicenter study investigating challenges associated with AC in all African countries is recommended. Despite such limitations, the current review can be a useful addition to understanding the state of neurosurgery and AC in Africa.

## CONCLUSION

Despite the existence of various constraints to its full uptake, the current review suggests a very encouraging prospect for the feasibility of AC in Africa because the absence of infrastructure has not prevented AC from being performed in many of the LMIC therein. Although the lack of technologies such as DES and neuroimaging devices can be considered a hindrance, they should not prevent neurosurgical centers from the uptake of AC. Similarly, patients in Africa should not be denied the benefits of AC procedures. Although encouraging, various challenges exist in practicing AC in Africa. More resources and training should be allocated to reduce disparity in neurosurgical care in Africa. Failure to address such gaps in neurosurgical treatment can result in wider disparities between developed and LMIC. Other low-resource neurosurgical centers in Africa are encouraged to safely incorporate AC into their neurosurgery programs. The goal should be to foster skills required to sustain AC within the host institutions and develop the next generation of neurosurgeons to change the paradigm.

## Supplementary Material

## SUPPLEMENTAL DIGITAL CONTENT

**Supplementary Table 1.** An Overview of Search Terms Used for Each Database. **Supplementary Table 2.** A Summary of Studies Reviewed. AC, awake craniotomy; COVID-19, coronavirus disease 2019; GA, general anesthesia. **Supplementary Table 3.** A Summary of Studies Characteristics. AC, awake craniotomy; SD, standard deviation; MAC, monitored anesthesia care; Min, minutes; NS, not specified; OR, operating room; SD, standard deviation. *The study stated “awake-asleep-awake” technique, which does not conform with conventional AC protocols where the mapping phase is conducted while the patient is awake. **Supplementary Table 4.** An Overview of Preoperative Details. AC, awake craniotomy; ASA, American Society of Anesthesiologists; BMI, body mass index; COPD, chronic obstructive pulmonary disease; CT, computerized tomography; DTI, diffusion tensor imaging; fMRI, functional magnetic resonance imaging; GA, general anesthesia; KPS; Karnofsky performance status; MMSE, mini-mental state examination; MRI, magnetic resonance imaging; NS, not specified; SD, standard deviation; STAI, State-Trait anxiety inventory. **Supplementary Table 5.** Intraoperative Details of AC. AC, awake craniotomy; DES; direct cortical stimulation; GA, general anesthesia; ICP, intracranial pressure; NS, not specified.
