# Supplementary material for: Awake Craniotomy in Africa: A Scoping Review of Literature and Proposed Solutions to Tackle Challenges
Source: Neurosurgery. 2023 Mar 24;93(2):274–91. doi: 10.1227/neu.0000000000002453 (PMC10319364; doi:10.1227/neu.0000000000002453)
Supplement: Supplementary file 1 [file neu-93-274-s001.docx]

**Supplementary Table 1.** An Overview of Search Terms Used for Each Database

| **Database** | **Search Results** | **Search Terms** |
| --- | --- | --- |
| **PubMed** | 3697 | (("awake"[All Fields] OR "awakeness"[All Fields] OR "awakes"[All Fields] OR "awaking"[All Fields]) AND ("craniotomy"[MeSH Terms] OR "craniotomy"[All Fields] OR "craniotomies"[All Fields])) OR (("awake"[All Fields] OR "awakeness"[All Fields] OR "awakes"[All Fields] OR "awaking"[All Fields]) AND ("brain"[MeSH Terms] OR "brain"[All Fields] OR "brains"[All Fields] OR "brain s"[All Fields]) AND ("surgery"[MeSH Subheading] OR "surgery"[All Fields] OR "surgical procedures, operative"[MeSH Terms] OR ("surgical"[All Fields] AND "procedures"[All Fields] AND "operative"[All Fields]) OR "operative surgical procedures"[All Fields] OR "general surgery"[MeSH Terms] OR ("general"[All Fields] AND "surgery"[All Fields]) OR "general surgery"[All Fields] OR "surgery s"[All Fields] OR "surgerys"[All Fields] OR "surgeries"[All Fields])) OR (("awake"[All Fields] OR "awakeness"[All Fields] OR "awakes"[All Fields] OR "awaking"[All Fields]) AND ("neurosurgery"[MeSH Terms] OR "neurosurgery"[All Fields] OR "neurosurgeries"[All Fields] OR "neurosurgery s"[All Fields] OR "neurosurgical procedures"[MeSH Terms] OR ("neurosurgical"[All Fields] AND "procedures"[All Fields]) OR "neurosurgical procedures"[All Fields])) OR (("awake"[All Fields] OR "awakeness"[All Fields] OR "awakes"[All Fields] OR "awaking"[All Fields]) AND ("brain mapping"[MeSH Terms] OR ("brain"[All Fields] AND "mapping"[All Fields]) OR "brain mapping"[All Fields]) AND ("awake"[All Fields] OR "awakeness"[All Fields] OR "awakes"[All Fields] OR "awaking"[All Fields]) AND ("cysts"[MeSH Terms] OR "cysts"[All Fields] OR "cyst"[All Fields] OR "neurofibroma"[MeSH Terms] OR "neurofibroma"[All Fields] OR "neurofibromas"[All Fields] OR "tumor s"[All Fields] OR "tumoral"[All Fields] OR "tumorous"[All Fields] OR "tumour"[All Fields] OR "neoplasms"[MeSH Terms] OR "neoplasms"[All Fields] OR "tumor"[All Fields] OR "tumour s"[All Fields] OR "tumoural"[All Fields] OR "tumourous"[All Fields] OR "tumours"[All Fields] OR "tumors"[All Fields]) AND ("resect"[All Fields] OR "resectability"[All Fields] OR "resectable"[All Fields] OR "resectates"[All Fields] OR "resected"[All Fields] OR "resecting"[All Fields] OR "resection"[All Fields] OR "resectional"[All Fields] OR "resectioned"[All Fields] OR "resectioning"[All Fields] OR "resections"[All Fields] OR "resective"[All Fields] OR "resects"[All Fields])) OR (("craniotomy"[MeSH Terms] OR "craniotomy"[All Fields] OR "craniotomies"[All Fields]) AND ("awake"[All Fields] OR "awakeness"[All Fields] OR "awakes"[All Fields] OR "awaking"[All Fields])) |
| **Scopus** | 3027 | ALL ( awake AND craniotomy OR awake AND brain AND surgery OR awake AND neurosurgery OR awake AND brain AND mapping AND awake AND tumour AND resection OR craniotomy AND while AND awake ) |
| **Web of Science** | 3468 | ALL=(awake craniotomy OR awake brain surgery OR awake neurosurgery OR awake brain mapping OR awake tumour resection OR craniotomy while awake) |
