# Supplementary material for: Awake Craniotomy in Africa: A Scoping Review of Literature and Proposed Solutions to Tackle Challenges
Source: Neurosurgery. 2023 Mar 24;93(2):274–91. doi: 10.1227/neu.0000000000002453 (PMC10319364; doi:10.1227/neu.0000000000002453)
Supplement: Supplementary file 2 [file neu-93-274-s002.docx]

**Supplementary Table 2.** A Summary of Studies Reviewed

| **Study** | **Article title** | **Journal** | **Country** | **Objective** |
| --- | --- | --- | --- | --- |
| **Mohamed et al., 2008**^36^ | Initial experience with awake craniotomy in Sudan | Sudan Journal of Medical Sciences | Sudan | To present the first experience with AC done with scalp block and sedation with unsupported airway in Sudan. |
| **Ali et al., 2009**^22^ | Awake craniotomy versus general anesthesia for managing eloquent cortex low-grade gliomas | Neurosciences | Egypt | To compare AC versus GA for excision of low-grade glioma in eloquent areas. |
| **Abdou et al., 2010**^23^ | Preliminary evaluation of ketofol-based sedation for awake craniotomy procedures | Egyptian Journal of Anaesthesia | Egypt | To evaluate the clinical efficiency of a ketofol-based sedation procedure during AC for varied surgical indications. |
| **Aboeldahab et al., 2011**^24^ | Is the usage of mannitol mandatory in awake craniotomy? A comparative study | Egyptian Journal of Anaesthesia | Egypt | To assess the value of using mannitol for the reduction of intracranial pressure and optimising surgical condition during AC. |
| **Mohamed et al., 2013**^37^ | Awake craniotomy, an unusual indication | Journal of Neurology and Neuroscience | Sudan | To assess the effectiveness of AC as an alternative anesthetic technique to GA in patients. |
| **Idowu et. al, 2016**^30^ | Awake craniotomy for intracranial lesions: An audit of the anaesthetists’ initial experience at the University College Hospital, Ibadan | Egyptian Journal of Anaesthesia | Nigeria | To describe and evaluate the experience of AC in a developing country institution. |
| **Meziane et. al, 2017**^38^ | Anaesthetic management for awake craniotomy in brain glioma resection: initial experience in Military Hospital Mohamed V of Rabat | Pan African Medical Journal | Morocco | To describe the institution's first experience with anesthetic management for AC. |
| **Elbakry et al., 2017**^25^ | Propofol-dexmedetomidine versus propofol-remifentanil conscious sedation for awake craniotomy during epilepsy surgery | Minerva Anestesiologica | Egypt | To evaluate the effectiveness of propofol - dexmedetomidine versus propofol - remifentanil conscious sedation during AC for epilepsy. |
| **Waly et al., 2018**^26^ | Quasi-experiment as an initial experience for conscious sedation in awake craniotomy: dexmedetomidine versus midazolam | Research and Opinion in Anesthesia & Intensive Care | Egypt | To compare the efficacy and safety of dexmedetomidine with midazolam during sedation of AC patients. |
| **Balogun et al., 2019**^31^ | Challenging the myth of outpatient craniotomy for brain tumor in a Sub-Saharan African setting: A case series of two patients in Ibadan, Nigeria | Surgical Neurology International | Nigeria | To assess the feasibility of AC by reporting two cases, highlighting the possible benefits of the procedure and some of the challenges posed by the practice environment in sub-Saharan Africa. |
| **Okunlola et al., 2019**^32^ | Challenges and prospects of awake Craniotomy in a resource-poor setting | International Journal of Neurology and Brain Disorders | Nigeria | To document the challenges and prospects of AC in a resource-poor setting. |
| **Benyaich et al., 2020**^39^ | Awake craniotomy with functional mapping for glioma resection in a limited -resource-setting: preliminary experience from a lower-middle income country | World Neurosurgery | Morocco | To assess the feasibility, safety, and efficiency of glioma resection using AC with minimal facilities in a limited-resource institution. |
| **Labuschagne et al., 2020**^40^ | Awake craniotomy in a child: assessment of eligibility with a simulated theatre experience | Case Reports in Anesthesiology | South Africa | To devise a simulated surgical experience attempt to assess the ability of a pediatric patient to undergo AC. |
| **Nasr et al., 2020**^27^ | Scalp block for awake craniotomy: Lidocaine- bupivacaine versus lidocaine-bupivacaine with adjuvants | Egyptian Journal of Anaesthesia | Egypt | To detect the safest and most effective adjuvants to be added to local anesthetic mixture used for scalp block during AC. |
| **Okunlola et al., 2020**^33^ | Awake craniotomy in neurosurgery: Shall we do it more often? | Interdisciplinary Neurosurgery | Nigeria | To investigate the feasibility of AC in lesions other than diffuse glioma. |
| **Okunlola 2021**^34^ | Awake craniotomy in a Covid-19 positive patient: The challenges and outcome | Interdisciplinary Neurosurgery | Nigeria | To report challenges and outcome of AC in a COVID-19 positive patient. |
| **Okunlola et al., 2021**^35^ | Parasagittal cystic meningioma mimicking hemangioblastoma: A case report | Surgical Neurology International | Nigeria | To investigate the feasibility of AC for resection of cystic meningioma. |
| **Abdelhameed et al., 2021**^28^ | Awake surgery for lesions near eloquent brain under scalp block and clinical monitoring: experience of single center with limited resources | The Egyptian Journal of Neurology, Psychiatry and Neurosurgery | Egypt | To evaluate the safety, feasibility, and outcome of AC in eloquent areas lesions. |
| **Morsy et al., 2021**^29^ | Predictors of stimulation-induced seizures during perirolandic glioma resection using intraoperative mapping techniques | Surgical Neurology International | Egypt | To report the incidence and predictors of stimulation-induced seizures during brain mapping under AC or GA. |

AC, awake craniotomy; COVID-19, coronavirus disease 2019; GA, general anesthesia.
