# Supplementary material for: Awake Craniotomy in Africa: A Scoping Review of Literature and Proposed Solutions to Tackle Challenges
Source: Neurosurgery. 2023 Mar 24;93(2):274–91. doi: 10.1227/neu.0000000000002453 (PMC10319364; doi:10.1227/neu.0000000000002453)
Supplement: Supplementary file 3 [file neu-93-274-s003.docx]

**Supplementary Table 3.** A Summary of Studies Characteristics

| **Study** | **Study period** | **Study design** | **Single/ multi- center** | **Condition treated** | **AC protocol** | **Primary sedation and analgesic** | **Mean AC operation time ± SD (range)** | **Follow-up** |
| --- | --- | --- | --- | --- | --- | --- | --- | --- |
| **Mohamed et al., 2008**^36^ | NS | Retrospective (case report) | Single | Tumor | Awake-awake-awake | Propofol and fentanyl | 2.5 hours | NS |
| **Ali et al., 2009**^22^ | Jan 2007 - Nov 2008 | Prospective | Single | Tumor | Asleep-awake-? | Propofol, fentanyl, atracurium, lidocaine, isoflurane | 173 ± 13 min | 6 months |
| **Abdou et al., 2010**^23^ | NS | Prospective | Single | Tumor | NS | Propofol. ketamine, and lidocaine with adrenaline | 168.8 ± 19.4 min | NS |
| **Aboeldahab et al., 2011**^24^ | 2006 - 2009 | Prospective | Single | Tumor and epilepsy | Awake-awake-awake | Propofol, fentanyl, bupivacaine, lidocaine, and adrenaline | 230.5 - 237 min | NS |
| **Mohamed et al., 2013**^37^ | 2008 - 2012 | Prospective | Single | Tumor | Awake-awake-awake | Propofol, fentanyl, bupivacaine | 139.25 ± 25.86 min | NS |
| **Idowu et. al, 2016**^30^ | Nov 2011 - Jan 2015 | Prospective | Single | Tumor and hematoma | Asleep-awake-asleep, asleep-awake-awake, awake-asleep-awake* | Propofol, fentanyl, bupivacaine, lidocaine with adrenaline | 184 ± 45.19 min | 6 months |
| **Meziane et. al, 2017**^38^ | NS | Retrospective (case report) | Single | Tumor | Awake-awake-asleep with MAC | Propofol, remifentanil, bupivacaine, and lidocaine | NS | NS |
| **Elbakry et al., 2017**^25^ | NS | Prospective | Single | Epilepsy | NS | Propofol and dexmedetomidine or propofol and remifentanil with bupivacaine, lidocaine, and epinephrine | 3.3 ± 1.3 hours | NS |
| **Waly et al., 2018**^26^ | Jan 2015 - Jan 2017 | Prospective | Single | Tumor | Awake-awake-awake | Propofol, fentanyl, lidocaine, and bupivacaine | 228 - 231 min | NS |
| **Balogun et al., 2019**^31^ | NS | Retrospective (case report) | Single | Tumor | Asleep-awake-asleep | Propofol / dexmedetomidine, fentanyl, bupivacaine and epinephrine | About 4 hours | 6 weeks to 8 months |
| **Okunlola et al., 2019**^32^ | July 2018 | Retrospective (case report) | Single | Tumor | Awake-awake-awake | Propofol, bupivacaine, xylocaine with adrenaline | About 3 hours | 5 months |
| **Benyaich et al., 2020**^39^ | Jun 2012 - Oct 2015 | Retrospective | Single | Tumor | Asleep- awake-asleep | Propofol and remifentanil, lidocaine, and adrenaline | 5 ± 15 hours (3-8) (OR time) | 6 months- 1 year |
| **Labuschagne et al., 2020**^40^ | NS | Retrospective (case report) | Single | Tumor | Asleep-awake-asleep | Propofol remifentanil, and dexmedetomidine | 77 min (awake phase) | 6 weeks |
| **Nasr et al., 2020**^27^ | Jan 2016 - Sep 2018 | Prospective | Single | Tumor | Asleep-awake-awake | Propofol, fentanyl, and dexmedetomidine | 203.8 - 208.2 min | NS |
| **Okunlola et al., 2020**^33^ | 18 months (NS) | Prospective | Single | Tumor, abcess, hematoma, foreign object removal | NS | Propofol, xylocaine, adrenaline, and bupivacaine | 1 - 2 hours and 40 min | NS |
| **Okunlola 2021**^34^ | NS | Retrospective (case report) | Single | Tumor | NS | Propofol, xylocaine, adrenaline, and bupivacaine | 2 hours | 30 days |
| **Okunlola et al., 2021**^35^ | NS | Retrospective (case report) | Single | Tumor | NS | NS | NS | 6 months |
| **Abdelhameed et al., 2021**^28^ | Jan 2015 - Jun 2018 | Retrospective | Single | Tumor | Awake-awake-awake | Propofol and fentanyl | 210 min ± 25.33 (170 - 250) | 3 months |
| **Morsy et al., 2021**^29^ | 2014 - 2019 | Retrospective | Single | Tumor | Awake-awake-awake | Propofol, dexmedetomidine, and fentanyl | NS | 3 months |

AC, awake craniotomy; SD, standard deviation; MAC, monitored anesthesia care; Min, minutes; NS, not specified; OR, operating room; SD, standard deviation. *The study stated “awake-asleep-awake” technique, which does not conform with conventional AC protocols where the mapping phase is conducted while the patient is awake.
