# Supplementary material for: Awake Craniotomy in Africa: A Scoping Review of Literature and Proposed Solutions to Tackle Challenges
Source: Neurosurgery. 2023 Mar 24;93(2):274–91. doi: 10.1227/neu.0000000000002453 (PMC10319364; doi:10.1227/neu.0000000000002453)
Supplement: Supplementary file 4 [file neu-93-274-s004.docx]

**Supplementary Table 4.** An Overview of Preoperative Details

| **Study** | **Preoperative localization method** | **Extent of lesion resection** | | **Hospitalization length days (mean) ± SD (range)** | | **Preoperative neurological signs and symptoms (n, %)** | **Inclusion criteria** | **Exclusion criteria** |
| --- | --- | --- | --- | --- | --- | --- | --- | --- |
|  |  | **Total/ near total n (%)** | **Subtotal n (%)** | **AC** | **GA** |  |  |  |
| **Mohamed et al., 2008**^36^ | MRI | NS | NS | 4 | NA | Dysphasia, left-sided facial palsy and memory loss (1, 100%) | The cardiac function of the patient made surgery under GA a potential risk. | NS |
| **Ali et al., 2009**^22^ | CT and MRI with contrast | 8 (40%) | 12 (60%) | 3.8 ± 4.15 | 8.15 ± 6.5 | NS | Patients aged between 20-55 years, ASA grade 1 and 2, undergoing resection of low-grade glioma in an eloquent brain area. | Confusion, communication difficulties, extreme anxiety, morbid obesity, COPD, complicated airway, end-organ failure, allergy to anesthetics or drugs used, non-supine surgeries and/or lasting more than 4 hours. |
| **Abdou et al., 2010**^23^ | NS | NS | NS | 3.1 ± 1.1 | NA | Headache (22, 78.6%), motor deficits (9, 32.1%), seizure (8, 64.3%) | Cooperative patients and physically able to tolerate AC. | Morbid obesity, COPD, complicated airway, allergy to anesthetics or drugs used, confusion, communication difficulties, extreme anxiety, tumors with involving significant dural invasion, non-supine position and/or lasting more than 4 hours. |
| **Aboeldahab et al., 2011**^24^ | NS | NS | NS | NS | NA | Seizure (23), headache (15), motor deficits (7) | Physically able to tolerate AC for either epilepsy or small sized tumors with minimal brain oedema located in eloquent brain areas. | BMI>30, communication difficulties, difficult airway, respiratory problems, non-supine surgery, high ICP, hydrocephalus or severe mid-line shift >1 cm). |
| **Mohamed et al., 2013**^37^ | MRI | NS | NS | NS | NA | NS | Variable co-morbidities that represent a constant threat to life if tumor resection is performed under GA. | NS |
| **Idowu et. al, 2016**^30^ | CT or MRI | NS | NS | 7 (median) | NA | Seizure (10, 55.6%), headache (8, 44.4%), hemiparesis (7, 38.8%), altered sensorium (3, 16.6%) and facioparesis (1, 5.5%) | Suitability was determined by the neurosurgical and anesthetic teams. Patients’ consent and age were considered. | NS |
| **Meziane et. al, 2017**^38^ | NS | NS | NS | NS | NA | Confusion (1, 100%) | Normal without any predicted difficulties for tracheal intubation or mask ventilation, and no cardiac or respiratory abnormalities. | NS |
| **Elbakry et al., 2017**^25^ | NS | NS | NS | NS | NA | NS | NS | Patients with allergy to any of the drugs used, alcohol and substance abuse, pregnancy, mental instability, morbid obesity and liver, kidney or cardiac comorbidities. |
| **Waly et al., 2018**^26^ | MRI | NS | NS | NS | NS | NS | Cooperative patients of both sexes, aged 21–65 years, and ASA grade I or II. | KPS< 60, MME< 24, severe language deficits, STAI>55, preoperative severe motor deficits, “large tumors more than 2 cm of midline shift, tumors with dural invasion, surgeries requiring positioning other than supine or with expected operative time of more than 4 h, BMI> 35, severe cardiac or respiratory disease, anticipated difficult-to-manage airway, history of allergy to medications used”, known alcohol or substance abuse, or refusal. |
| **Balogun et al., 2019**^31^ | MRI | 2 (100%) | 0 | 1 | NA | Headache, vomiting, progressive bilateral visual loss, and right-sided weakness (1, 50%), headache and altered consciousness (1, 50%) | According to the criteria suggested by Carrabba et al. (2008)^81^. | NS |
| **Okunlola et al., 2019**^32^ | NS | 2 (100%) | 0 | 5-8 days | NA | Headache and hemiparesis (2, 100%) | NS | NS |
| **Benyaich et al., 2020**^39^ | MRI | 8 (40%) | 12 (60%) | 4.5 days (range, 2-11) | NA | Seizure (14, 70%), intracranial hypertension (6, 30%), memory disorders with mild dysphasia (1, 5%), right hemiparesis (1, 5%), left facial palsy (1, 5%) | “Based on typical radiologic images of supratentorial diffuse low-grade glioma close to or within an eloquent area.” | Patients with severe neurologic deficits, uncooperativeness, KPS<70%, severe medical comorbidities, American Society of Anesthesiologists score ≥ 3, and paediatrics. |
| **Labuschagne et al., 2020**^40^ | MRI | 1 (100%) | NA | NS | NA | Seizure and motor deficits | NS | NS |
| **Nasr et al., 2020**^27^ | NS | NS | NS | NS | NA | NS | NS | NS |
| **Okunlola et al., 2020**^33^ | CT and MRI | NS | NS | 5-11 days | NA | Headache (7), complex partial seizure (1), righ hemiparesis (3), left hemiplegia (1), left hemiparesis (1), receptive aphasia (1) | “Awake and cooperative patients with suitable lesion location in the brain convexity whose surgery were expected to last not more than four hours.” | NS |
| **Okunlola 2021**^34^ | MRI | 1 (100%) | 0 | NS | NA | Recurrent seizure, right hemibody weakness | NS | NS |
| **Okunlola et al., 2021**^35^ | MRI | 1 (100%) | 0 | NS | NA | Seizure, headache, right hemibody weakness, motor aphasia (1, 100%) | NS | NS |
| **Abdelhameed et al., 2021**^28^ | CT, MRI with contrast, and MRI tractography | 15 (75%) | 4 (20%) (1 biopsy, 5%) | 2.7 ± 2.67 (2-14) | NA | Motor weakness (8, 40%), headache (6, 30%), seizure (6, 30), speech difficulty (3, 15%) | Patients with supratentorial lesions related to motor or language areas. | Infratentorial lesions, uncooperative (children and psychiatric patients), and high BMI with expected obstructive sleep apnea. |
| **Morsy et al., 2021**^29^ | MRI with contrast, fMRI and DTI | 32 (80%) | 8 (20%) | NS | NS | Headache (30, 75%), seizure (19, 47.5%), motor weakness (18, 45%), sensory dysfunction (16, 40%), cognitive deficit (6, 15%), dysarthria (10, 25%) | 70 >Age >18 without major cardiopulmonary co-morbidities, fluent in speaking and understanding without preoperative cognitive impairment (MMSE> 24), no severe language or motor deficits or anxiety. | NS |

AC, awake craniotomy; ASA, American Society of Anesthesiologists; BMI, body mass index; COPD, chronic obstructive pulmonary disease; CT, computerized tomography; DTI, diffusion tensor imaging; fMRI, functional magnetic resonance imaging; GA, general anesthesia; KPS; Karnofsky performance status; MMSE, mini-mental state examination; MRI, magnetic resonance imaging; NS, not specified; SD, standard deviation; STAI, State-Trait anxiety inventory.
