# Supplementary material for: Awake Craniotomy in Africa: A Scoping Review of Literature and Proposed Solutions to Tackle Challenges
Source: Neurosurgery. 2023 Mar 24;93(2):274–91. doi: 10.1227/neu.0000000000002453 (PMC10319364; doi:10.1227/neu.0000000000002453)
Supplement: Supplementary file 5 [file neu-93-274-s005.docx]

**Supplementary Table 5.** Intraoperative Details of AC

| **Study** | **Surgical position** | **Eloquent area lesion (n, %)** | **Intraoperative eloquent area mapping using DES** | **Direct electrical stimulation detail** | **Intraoperative complications (n, %)** | **Conversion to GA (n, %)** |
| --- | --- | --- | --- | --- | --- | --- |
| **Mohamed et al., 2008**^36^ | Left lateral | NS | No- continuous monitoring of patient’s speech and motor | Not used | None | None |
| **Ali et al., 2009**^22^ | Supine | Motor and language | No- continuous monitoring of patient’s speech and motor | Not used | Over-sedation (5, 12.5%), increased ICP (4, 10.0%), focal seizure (2, 5.0%), agitation (2, 5.0%), severe pain (2, 5.0%) | None |
| **Abdou et al., 2010**^23^ | Supine | NS | NS | NS | Heart rate variability (4, 14.2%), blood pressure changes (3, 10.7%), focal seizure (2, 7.1%), respiratory depression (2, 7.1%), peripheral blood desaturation with SpO2 <90% (1, 3.6%), nausea (1, 3.6%) | None |
| **Aboeldahab et al., 2011**^24^ | Supine | NS | NS | NS | Nausea (4, 10.0%), seizure (2, 5.0%) | None |
| **Mohamed et al., 2013**^37^ | Supine | NS | No- continuous monitoring of patient’s speech and motor | Not used | None | None |
| **Idowu et. al, 2016**^30^ | NS | NS | NS | NS | Hypertension (3, 16.7%), tachycardia (3, 16.7%), hypotension (1, 12.5%), focal seizure (1, 12.5%) | None |
| **Meziane et. al, 2017**^38^ | Right lateral | Motor and language | NS | NS | None (0, 0%) | None |
| **Elbakry et al., 2017**^25^ | NS | NS | NS | NS | Nausea (6, 10.0%), vomiting (3, 5.0%), respiratory depression (3, 5.0%), hypotension (7, 11.7%), hypertension (4, 6.7%), oxygen desaturation (3, 5.0%) | 5 |
| **Waly et al., 2018**^26^ | Supine | Sensory, motor and language | Yes | NS | Seizure (3, 12.5%) | 1 |
| **Balogun et al., 2019**^31^ | Supine | NS | No | Not used | None (0, 0%) | None |
| **Okunlola et al., 2019**^32^ | NS | Motor | No | Not used | None (0, 0%) | None |
| **Benyaich et al., 2020**^39^ | Supine or lateral | Motor and language | Yes- motor, sensory, and language | “A bipolar electrode connected to a generator delivering a biphasic current at a frequency of 60 Hz with a pulse phase duration of 1 millisecond.” 1 mA amplitude initiation and gradual increase by “0.5 mA until a response was obtained, without exceeding 6 mA.” “Each site was stimulated for 1-2 seconds for motor or sensory functions and 3-4 seconds for speech testing.” | Seizure (5, 25.0%), transfusion (1, 5.0%), sudden right hemiplegia (1, 5.0%), deep asthenia (1, 5.0%) | None |
| **Labuschagne et al., 2020**^40^ | Semilateral | Motor (1, 100%) | Yes- motor and language | “A bipolar electrode, delivering a biphasic current, was applied, and positive and negative motor and speech mapping was performed. A threshold of 1 mA was used to limit surgical resection.” | NS | None |
| **Nasr et al., 2020**^27^ | NS | NS | Yes | NS | None | None |
| **Okunlola et al., 2020**^33^ | NS | NS | NS | NS | Excessive hemorrhage (1, 12.5%) | None |
| **Okunlola 2021**^34^ | NS | Motor (1, 100%) | NS | NS | None (0, 0%) | None |
| **Okunlola et al., 2021**^35^ | NS | NS | NS | NS | None (0, 0%) | None |
| **Abdelhameed et al., 2021**^28^ | Variable according to tumor location (NS) | Motor (11, 55%), language (9, 45%) | Yes- motor, sensory and language | A bipolar stimulator delivered biphasic waves in 4 s trains at 60 HZ at low current < 5 mA, with starting at 1.5 mA and increase at 0.5 m increments each time to a maximum of 5 mA. | Seizure (2, 10.0%) | 1 (5%) due to uncontrolled seizure |
| **Morsy et al., 2021**^29^ | NS | Motor | Yes | A bipolar stimulator delivered “50-60 Hz constant current biphasic square wave” with a “duration of 1–2 s per stimulation.” “Stimulation was started with 2 mA increased to a maximum (6–10).” Stimulation threshold for  cortical (4.9 mA ± 0.42) and subcortical (8.3 mA ± 0.62). | Seizure (4, 10.0%) | None |

AC, awake craniotomy; DES; direct cortical stimulation; GA, general anesthesia; ICP, intracranial pressure; NS, not specified.
